# Effect of Diclofenac and Simvastatin on Bone Defect Healing—An In Vivo Animal Study

**DOI:** 10.3390/biomimetics7040143

**Published:** 2022-09-25

**Authors:** Theodora Karanikola, Angeliki Cheva, Katia Sarafidou, Maria Myronidou-Tzouveleki, Ioannis Tsavdaridis, Eleana Kontonasaki, Anastasios Tsirlis

**Affiliations:** 1 Department of Oral Surgery, Implantology and Dental Radiology, School of Dentistry, Faculty of Health Sciences, Aristotle University of Thessaloniki (A.U.Th), 54124 Thessaloniki, Greece; 2 Pathology Department, School of Medicine, Faculty of Health Sciences, Aristotle University of Thessaloniki (A.U.Th), 54124 Thessaloniki, Greece; 3 Department of Prosthodontics, School of Dentistry, Faculty of Health Sciences, Aristotle University of Thessaloniki (A.U.Th), 54124 Thessaloniki, Greece; 4 1st Laboratory of Pharmacology, School of Health Sciences, Faculty of Medicine, Aristotle University of Thessaloniki, 56224 Thessaloniki, Greece

**Keywords:** diclofenac, simvastatin, bone healing, Wistar rats, histomorphometry

## Abstract

Non-steroidal, anti-inflammatory drugs and statins are two widely prescribed drug classes that affect bone formation. The aim of this study was to elucidate the effect of diclofenac and simvastatin in artificial bone defect healing. One hundred and forty-four male Wistar rats were used, and the specimens were divided into groups, with respect to the route of drug administration and the type of defect healing (with or without collagen membrane), and subgroups, with respect to the study duration (2, 4 or 8 weeks). Diclofenac was intramuscularly administered while simvastatin was administered both systemically and locally. Animals were euthanized and specimens were histomorphometrically analyzed to evaluate the percentage of new bone formation (%). Bone healing that occurred without any intervention developed more steadily than that of all other groups. Diclofenac exerted a clear, direct inhibitory effect on bone healing and its systemic administration should be avoided. The systemic administration of simvastatin was related to severe myopathy, while the solvent for the local administration of simvastatin seemed to play significant role in bone growth, as simvastatin, when it is administered intraperitoneally in a DMSO solution, appeared to promote bone healing. Local administration may have a significant impact on bone healing and it should be further investigated with the type of solvent or carrier that is used, which both may play a significant role in bone repair induction.

## 1. Introduction

Bone-repair mechanisms are of paramount importance in everyday clinical practice. The success of dental interventions such as tooth extraction, the insertion of dental implants or guided bone regeneration modalities, is correlated to sufficient bone healing. Bone healing, whether it is defect/wound healing or peripheral osteogenesis around implants, is a critical process, and any pharmacological intervention could be a catalyst for bone repair. It is noteworthy that many medications that are taken by patients to treat serious medical conditions can affect the quantity and quality of their bones. Moreover, the medication that is prescribed in order to reduce post-operative pain or decrease tissue swelling may affect the bone-healing mechanisms. Non-steroidal, anti-inflammatory drugs (NSAIDs) are most widely prescribed after dental interventions are performed [1,2]. On the other hand, a lot of dental patients receive statins, which are used to reduce the high levels of blood cholesterol.

Simvastatin is a statin that has been reported to have an osteoblast-stimulating effect. Mundy et al. [3] were the first to investigate a mouse calvaria defect model and to report that simvastatin could stimulate bone regeneration and promote bone formation. The positive effect of simvastatin with regards to osteoinduction and osteogenesis has also been reported by other investigators [4,5]. A recent meta-analysis stated that simvastatin shows a promising potential for alveolar bone regeneration in the optimal dose of 0.5–10 mg, depending on the route of its administration [6]. Tahamtan et al. [7] concluded in a large recent review that the systemic and, in particular, local application of statins possesses a beneficial effect on alveolar bone loss, the osseointegration of implants and wound/bone healing. However, there is controversy in the reported data regarding statin use and the longitudinal change in bone density that occurs [8]. In addition, statins have been associated with severe adverse effects, and they may in some cases inhibit bone resorption [9,10,11]. The controversies that arise in the literature may be attributed to the route of the administration as well as dose/dosage of the administered drug [12].

On the other hand, data concerning the effect of diclofenac—which is also often prescribed after dental interventions are performed—on bone formation are scarce in the literature. Diclofenac is classified among the anthranilic acid derivatives, and it has been associated with gastrointestinal, cardiovascular, hepatic, cerebral or pulmonary complications [13]. In dentistry, the administration of diclofenac has been reported to delay new bone formation after a tooth extraction has been performed [14]. Furthermore, diclofenac may inhibit dental movement during an orthodontic treatment [15]. The negative effect of diclofenac on bone healing may be attributed to its adverse effects, since it has been reported that diclofenac inhibits the proliferation and induces the apoptosis of human osteoblast cells [16]. Furthermore, on a cellular level, diclofenac may show a negative impact on pre-osteoblast cell growth [17]. However, recent studies have assumed the negative impact of diclofenac on bone metabolism to be subsidiary [18], while Cai et al. [19] reported that diclofenac did not adversely affect the osseointegration of dental implants and bone healing in the calvaria, neither in the short nor the long term. Based on the abovementioned studies, the aim of the present in vivo animal study was to elucidate the effect of diclofenac and simvastatin in bone defect healing, based on the drug administration route, in a rat in vivo model.

## 2. Materials and Methods

### 2.1. Animal Study Design

The present research was conducted by the Department of Dentoalveolar Surgery, Implantology and Oral Radiology, Faculty of Dentistry, at the Aristotle University of Thessaloniki. A special ethics permission was obtained from the Ethics Committee, School of Dentistry, AUTh, (registration number: 153/17-06-09) for the laboratory use of these animals. The study was performed on male Wistar rats, weighing 300–400 gr. The rats were housed at the institutional animal center in individual cages and fed with a standard laboratory ad libitum diet. The animal protocol was designed to minimize the pain or discomfort that they experienced, and it was conducted according to the guidelines that are defined by the Directorate of Veterinary Medicine of Central Macedonia (registration number: 153/17-06-09). According to a sample size calculation that was performed prior to experimentation, it was calculated that at least 8 defects would be needed per group in order to detect a difference of approximately 15% in the new bone formation between the groups, with a = 0.05 and 80% statistical power. With respect to the reduction principle (the replacement, reduction and refinement principles in animal research), we decided to create 2 calvarial defects per animal to avoid the compromise of the samples in each group in case of any animal death occurring or the destruction of the specimens during the conduction of the histologic analysis. In cases of animal death or the destruction of the specimens during the conduction of the histologic analysis, the animals were not replaced. For this reason, a diversity between the samples per group is observed. However, the minimum number of defects (n = 8) per group was maintained in order to perform a reliable statistical analysis. In total, 144 animals were used and two calvarial defects were induced in each animal, which resulted in 288 samples. Ninety-three of the samples could not be included in the results for the evaluation, mainly due to death of the animal or due to the destruction of the sample during the conduction of the histological analysis.

Specimens were divided into 7 main groups, according to the route of administration of the pharmacological agents and the type of defect healing (with or without a collagen membrane). They were also divided into 18 subgroups, with respect to the experiment time span that was either 2, 4 or 8 weeks. Thus, the following groups were formed:

A. A control group that is without any instances of us interfering in the natural defect healing;

B. Those with defect healing after the application of a collagen membrane (fleece type);

STS. Those who underwent the systemic administration (intraperitoneal injection) of a simvastatin (Sigma-Aldrich, Missouri, USA) solution (10 mg/kg of body weight daily) throughout the experiment;

STL. Those who underwent the local application of a collagen membrane (fleece type) that was impregnated with 0.1 mL of a simvastatin solution at a concentration of 5 mg/mL in water for it to be injected;

STDS. Those who underwent the systemic (intraperitoneal) administration of simvastatin (10 mg/kg of body weight) that was dissolved 50% in water and 50% in DMSO (Sigma-Aldrich, Missouri, USA) daily, throughout the experiment;

STDL. Those who underwent the local application of a collagen membrane (fleece type) that was impregnated in 0.1 mL of a simvastatin solution that was dissolved in DMSO at a concentration of 5 mg/mL;

DFS. Those who underwent a systemic diclofenac administration (intramuscular injection of diclofenac (Voltaren^®^, Novartis Hellas, Athens, Greece) 5 mg/kg of body weight daily throughout the experiment).

The groups’ classification and the sample size per group is given in Table 1.

Dimethylsulfoxide (DMSO) was used as a chemical solvent to provoke the better absorption of simvastatin [20]. The collagen membrane (BCG-25.5 BIOCOLLAGEN FLEECE 25 × 50 × H8 mm, Bioteck S.r.l., Visenza, Italy) that was presented at a 6 mm diameter and was produced from collagen type I and III from the calcaneal tendons of horses.

### 2.2. Surgical Procedure

**Figure 1:**
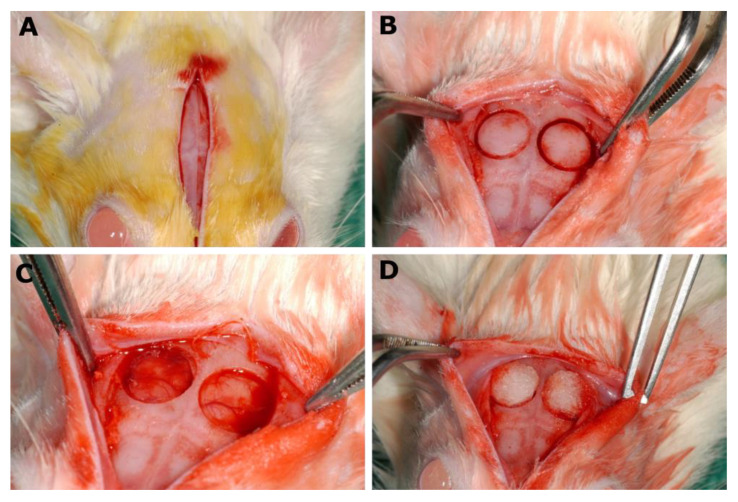
Pictures of the surgical interventions. (**A**) Incision along the sagittal midline of the cranium; (**B**) After the use of trephine bur; (**C**) Empty cavities; (**D**) Placement of the collagen membrane.

Every animal received an antibiotic prophylaxis (Begalin-P^®^ PD inj. Sol; Pfizer Hellas A.E., 50 mg/kg subcutaneously) 1 h before the general anesthesia (ketamine 20–30 mg/kg and xylazine 2–5 mg/kg intramuscularly), as well as after the surgery [21]. The surgical procedures were performed under a proper aseptic technique, and the skull of every animal was shaved, while the skin was disinfected with 10% povidone iodine solution. The parietal bones were exposed through an incision along the sagittal midline of the cranium (Figure 1A). Two circular cranial defects were created using a trephine bur, which were 6 mm in diameter on both sides of the sagittal suture (Figure 1B,C), using saline to prevent excessive heating. Special care was taken to avoid the damage of the dura matter of the Wistar rats. Then, depending on the group, the following procedure was performed: the suturing of the wound without any other intervention (group A and all groups related to the systemic administration of substances, groups DFS, STS and STDS), or the placement of the collagen membrane (group B and local application of collagen membrane, groups STL and STDL) (Figure 1D). The flaps were then sutured back in place, layer by layer, with non-resorbable suture.

The animals were euthanized by an intravenous administration of pentobarbital (18% solution, 60 mg/kg) after 2, 4 or 8 weeks, according to the protocol. Following their euthanasia, the cranial bone area containing the defects was block-sectioned for the histological preparation and histomorphometric analysis. At the same time, the samples of the brain, liver, kidney, and quadriceps muscle were taken from all of the rats, in order to detect any toxic side-effects that were caused by the administered medication.

### 2.3. Histological Preparation

All of the samples were initially immersed in 10% formaldehyde solution, followed by their immersion in EDTA solution (3–5 days). The standard histological procedure was followed. Paraffin sections of 3–5 μm were conventionally stained with eosin-hematoxylin. Afterwards, a paraffin block was created and histological sections of 3–5 μm thickness were taken. The sections were then conventionally stained with eosin-hematoxylin.

### 2.4. Histomorphometry

**Figure 2:**
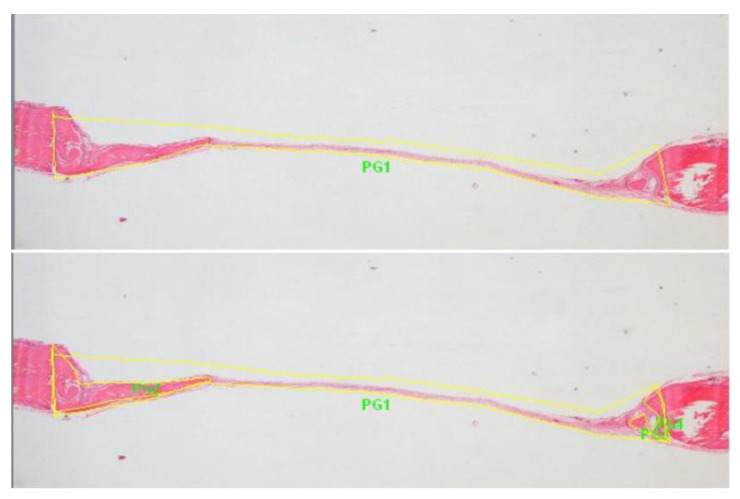
Pictures for histomorphometric measurements. PG1: total area (yellow) of bone defect, PG2, PG3 and PG4: areas of new bone formation within the total area of bone defect.

The samples were viewed under an optical microscope (ZeissAxio Lab Germany), and the development and degree of the maturation of the new bone was recorded. Digital images were captured (SONY DSC F707) to perform histomorphometric measurements using the special software Image Pro Plus (Media Cybernetics Inc., Rockville, MD, United States). In each bone defect, the 2 longest central sections were analyzed and measured, in which, the percentage of new bone (e.g., PG2, PG3, etc.) to the total area of the defect (PG1) was calculated, while the average of these values represented the final measurement of the newly formed bone (Figure 2).

### 2.5. Statistical Analysis

A statistical analysis was performed using SPSS Software (SPSS 19). The Mann–Whitney U test for independent samples was applied. In detail, a statistical analysis was performed in order to compare the percentage (%) of defect healing with respect to the experiment time span among: a. subgroups within each group in order to assess the difference in the percentage of defect healing among the samples of different experiment time spans, b. subgroups of different groups (with respect to the route of drug administration) in order to assess the differences in the bone healing percentage between specimens that came from the groups with the same experimental time span, and c. subgroups within different groups that experienced the same route of drug administration and study duration. Statistical significance was determined at the *p* < 0.05 level.

## 3. Results

**Figure 3:**
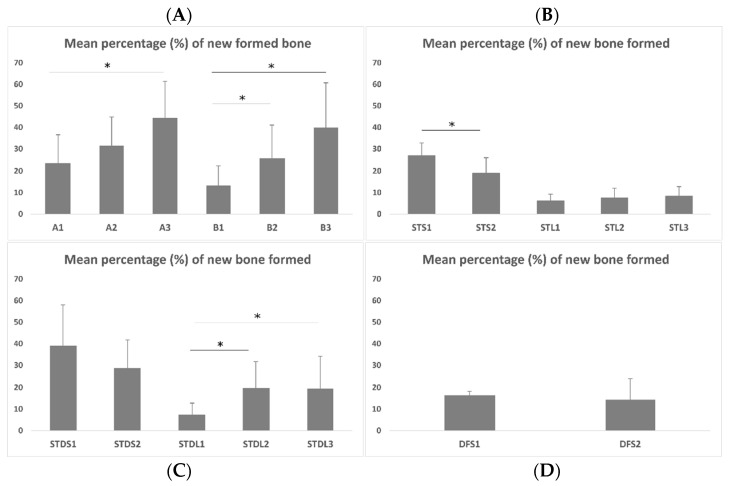
The average percentage (%) of newly formed bone in different groups. (**A**) Control vs. application of collagen membrane; (**B**) Systemic vs. local administration of simvastatin dissolved in water; (**C**) Systemic vs. local administration of simvastatin dissolved in DMSO; (**D**) Systemic (intramuscular) administration of diclofenac. * means statistically significant difference between the subgroups of each group (at different experimental time span).

**Figure 4:**
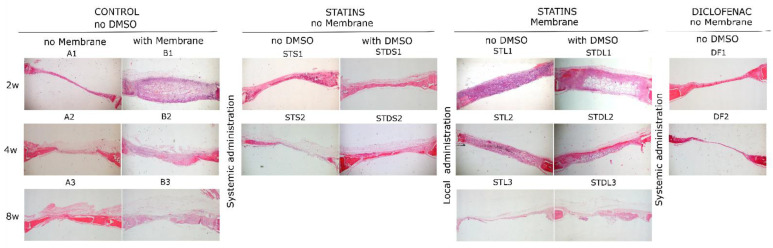
Histological micrographs of all of the groups showing the degree of new bone formation in the created bone defect for all of the subgroups. Magnification 20×.

The percentage (%) of newly formed bone per sample for different groups is given in Figure 3, and the histological micrographs from all of the groups are in Figure 4. In group A, the percentage of new bone formation presented statistically significant differences between the 2nd and 8th week (A1 vs. A3, *p* = 0.012), while in group B, the differences were statistically significant between the 2nd and 4th week (B1 vs. B2, *p* = 0.04) and between the 2nd and 8th week (B1 vs. B3, *p* = 0.001) (Figure 3A). In group STS, there was a statistically significant reduction after 4 weeks (STS1 vs. STS2, *p* = 0.007), while in the subgroups of the STL group, no statistically significant differences were observed (Figure 3B). In group STDS, the bone formation analysis did not present any significant difference, however, statistically significant differences were observed between the 2nd and 4th week (STDL1 vs. STDL2, *p* = 0.015) and between the 2nd and 8th week (STDL1 vs. STDL3, *p* = 0.019) of the STDL subgroups. No significant differences were observed between the subgroups where diclofenac was administered.

**Figure 5:**
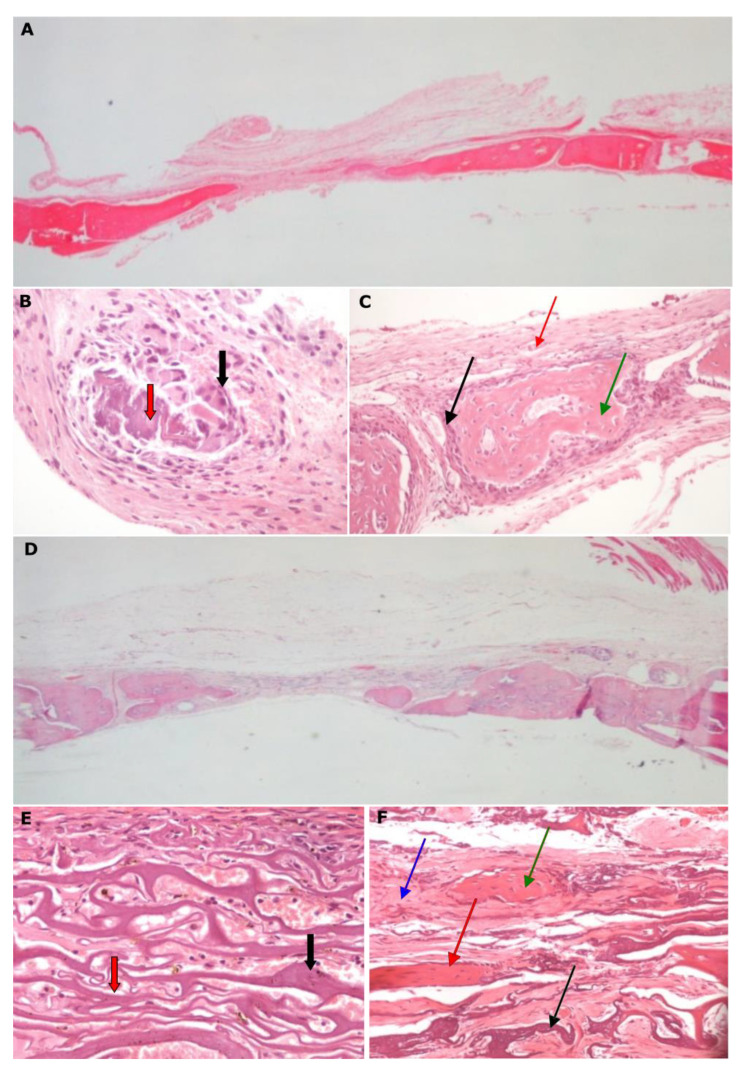
Histological micrographs of groups A and B. (**A**) Group A3, magnification 20×: ossification occurs/develops from periphery to center, which is occupied with fibrous connective tissue; (**B**) Group A1, magnification 400×: presence of osteoclasts during necrotic tissue resorption (red arrow); (**C**) Group A1, magnification 200×: newly formed osseous tissue (green arrow) surrounded by osteoblasts and a ring of fibrous connective tissue (red arrow); (**D**) Group B3, magnification 20×: membrane is completely degraded and new bone has formed from periphery to center, which is occupied with fibrous connective tissue; (**E**) Group B1, magnification 400×: presence of membrane (black arrow) without remarkable degradation of vasculature (red arrow); (**F**) Group B2, magnification 200×: extended membrane degradation (black arrow), newly formed osseous tissue (green arrow) surrounded by osteoblasts (red arrow) and growth of fibrous connective tissue (blue arrow).

In group A, there was a statistically significant difference in the bone healing percentages between the samples that were taken after 2 and 8 weeks (Figure 5A–C). As far as group B is concerned, the use of a collagen membrane affected, positively, the bone formation, with these results being statistically significant after 4 weeks and after 8 weeks of healing (Figure 5D–F).

**Figure 6:**
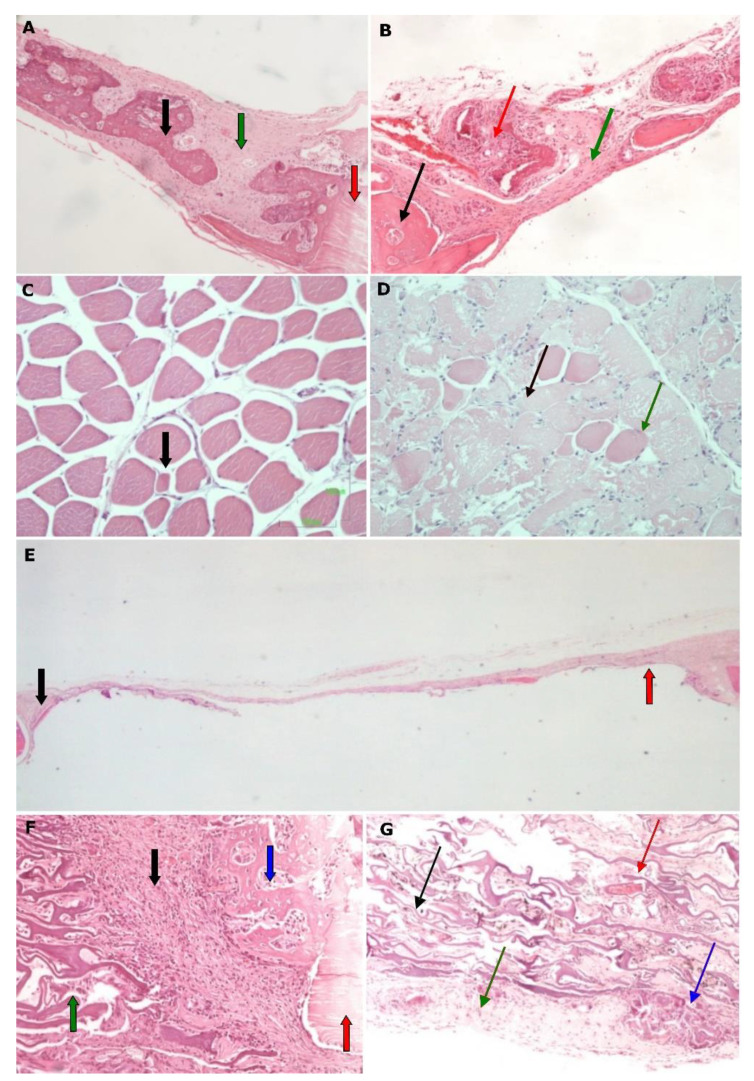
Histological micrographs of groups STS and STL. (**A**) Group STS1, magnification 100×: bone tissue with abundant new osseous tissue formation (black arrow) in contact with the margin of the defect (red arrow) and fibrous connective tissue growth (green arrow); (**B**) Group STS2, magnification 100×: the new tissue (black arrow) begins to degrade (red arrow) and is replaced by fibrous connective tissue (green arrow); (**C**) Group STS1, magnification 100×: microscopic image of quadriceps muscle with limited atrophic muscle fibers (black arrow); (**D**) Group STS2, magnification 100×: microscopic image of quadriceps with almost complete destruction of muscle fibers (black arrow), replaced by fibrous connective tissue and few remaining muscle fibers (green arrow); (**E**) Group STL3, magnification 20×: complete degradation of membrane and this has been replaced by thin fibrous connective tissue of limited thickness (red arrow). Minimal newly formed osseous tissue is observed at the margin of the defect (black arrow); (**F**) Group STL1, magnification 200×: at the margins of the defect (red arrow), the formation of new osseous tissue begins (blue arrow), the membrane is slightly stretched without significant signs of degradation (green arrow), while inflammatory infiltrate can be seen (black arrow); (**G**) Group STL2, magnification 200×: at 4 weeks, moderate degradation of the membrane (black arrow) is observed, marked by vessels (red arrow) and by its gradual replacement by fibrous connective tissue (green arrow). Additionally, material degradation is observed, with multinucleated foreign body-type giant cells being present (blue arrow).

Moreover, in the STS groups the systemic administration of simvastatin induced bone healing up to the second experimental week, but after this period, it resulted in a statistically significant bone loss (STS1 vs. STS2, *p* = 0.007) (Figure 6A,B). This finding suggests an initially positive, but then a negative osteogenic effect of simvastatin when it is administered systemically. This action may be considered to be indirect as a result of the intense myopathy that was observed in the histological preparations (Figure 6C,D), or due to the development of inflammation in the peritoneal region where the drug was injected. The local administration of simvastatin (STL groups) did not show any positive effect in bone healing, since bone formation remained stable and stopped after 2, 4 or 8 weeks of healing (Figure 6E–G).

**Figure 7:**
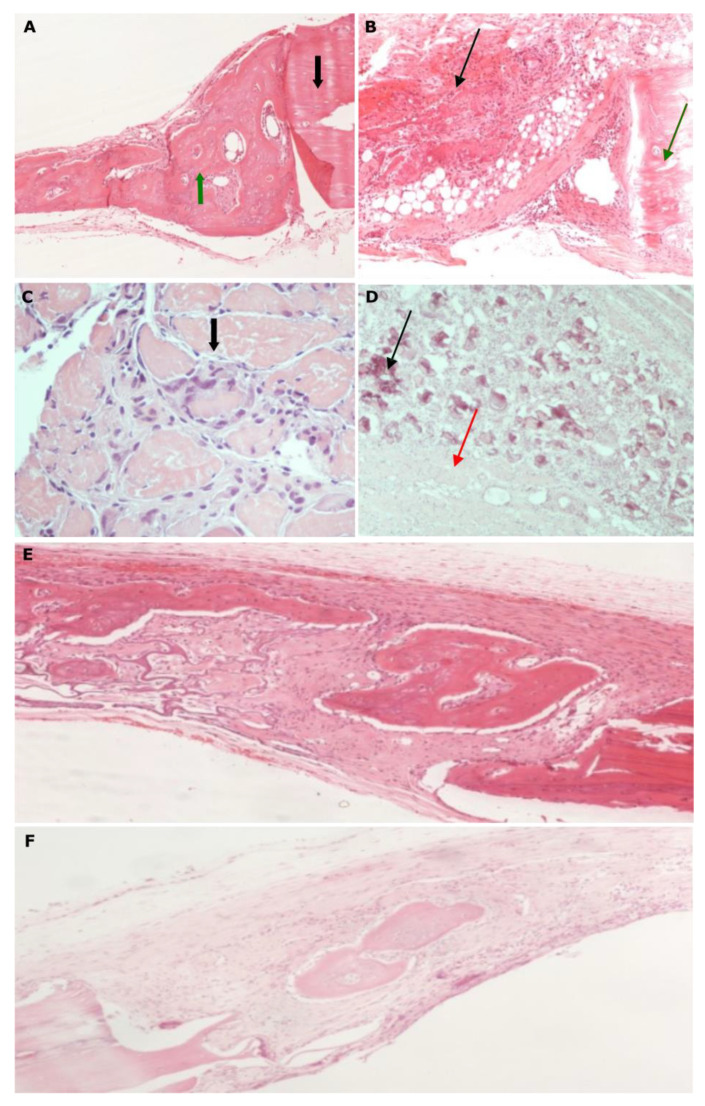
Histological micrographs of groups STDS and STDL. (**A**) Group STDS1, magnification 200×: microscopic image of bone tissue with abundant new bone formation (green arrow) broadly thick and in triangular shape (triangle base in contact with the defect margins (black arrow) and triangle apex towards the center of the defect); (**B**) Group STDS2, magnification 200×: extended degradation (black arrow) can be seen at the defect’s margins (green arrow) and adipose tissue formation (red arrow); (**C**) Group STDS1, magnification 100×: microscopic image of quadriceps muscle with extensive necrosis. Foreign body-type multinucleated giant cells are observed (black arrow); (**D**) Group STDS2, magnification 100×: microscopic image of quadriceps muscle with marked Ca^++^ deposition (black arrow) and extensive fibrosis (red arrow); (**E**) Group STDL2, magnification 20×: new osseous tissue develops, while the membrane has been degraded to a significant extent and replaced by fibrous connective tissue; (**F**) Group STDL3, magnification 20×: the new osseous tissue remains constant after 4 weeks, while the fibrous connective tissue is loose.

Furthermore, for the samples where the simvastatin was systemically administrated after its dissolution in water and DMSO (STDS groups), some healing bone activity was recorded until the 2nd week, while afterwards, bone loss was observed (Figure 7A–D). The recorded amount of bone loss was not statistically significant. For the STDL groups there was a statistically significant increase in bone regeneration between the 2nd (STDL1) and 4th (SDTL2) weeks (*p* ≤ 0.05), which remained stable between the 2nd (STDL1) and 8th (SDTL3) weeks (*p* ≤ 0, 05), while no changes in osteogenic activity were observed between the 4th (SDTL2) and 8th (SDTL3) weeks (Figure 7E,F).

**Figure 8:**
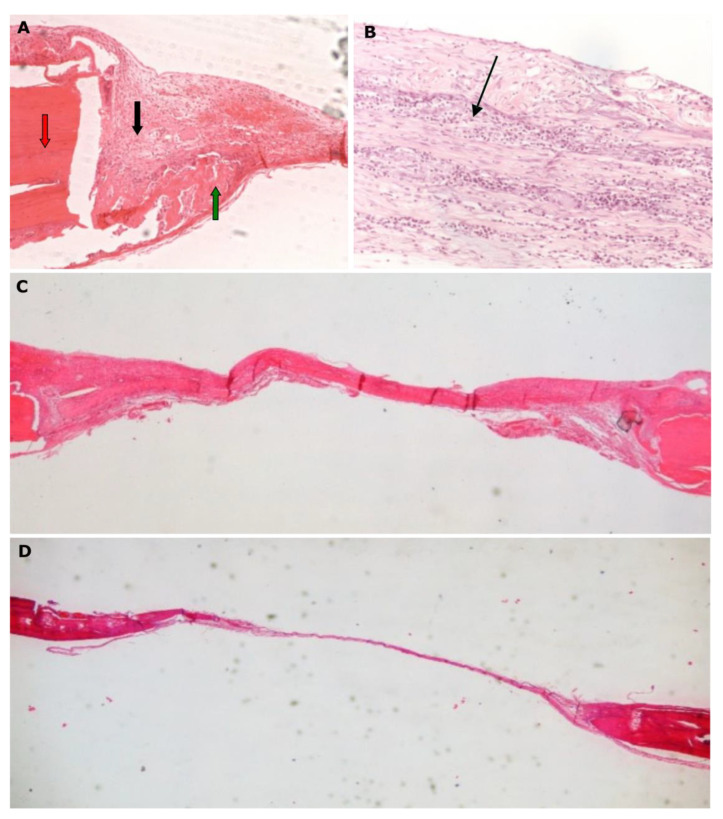
Histological micrographs of group DFS. (**A**) Group DFS1, magnification 200×: limited new bone (green arrow) develops at the margins of the defect (red arrow), while significant inflammation is also observed (black arrow); (**B**) Group DFS1, magnification 200×: development of extended inflammation of the fibrous connective tissue in the center of the defect (black arrow); (**C**) Group DFS1, magnification 20×:, growth of dense fibrous connective tissue; (**D**) Group DFS2, magnification 20×: limited new osseous tissue, loose fibrous connective tissue.

Finally, with regards to the DFS test groups, no bone healing—and to some extent a reduction of bone formation—was recorded, implicating that there is a negative effect of the systemic administration of diclofenac (Figure 8).

When we were performing a statistical analysis to compare the bone formation among subgroups of different groups with the same experimental time span, it was shown that subgroup B1 presented statistically inferior bone healing when it was compared to that of subgroup A1 (2 weeks). After the 4th and 8th week, the difference was not statistically significant (A2 vs. B2, and A3 vs. B3). The gradual resorption of the membrane at 4 and 8 weeks resulted in faster bone healing, reaching similar levels to that of the natural bone healing group. Moreover, there was a statistically significant difference in the bone formation both after 2 weeks (*p* < 0.001) and after 4 weeks (*p* = 0.002) between the groups STL and STS, suggesting that the intravenous administration of simvastatin is superior to its local application. Furthermore, when comparing the samples of the groups STDL and STDS, there was a statistically significant difference in bone healing, with the systemic administration of simvastatin resulting in better values when they are compared to those that were obtained by the local application of it through the collagen membrane after 2 weeks (*p* = 0.001), but not after 4 weeks (*p* = 0.137).

The recorded values between the groups with the same route of drug administration and same experimental time span revealed a statistically significant positive effect for the systemic administration of simvastatin that was dissolved in DMSO when it was compared to that of natural bone healing after 2 weeks (STDS1 vs. A1 *p* = 0.033) and when it was compared to that of the systemic diclofenac administration (STDS1 vs. DFS1, *p* < 0.001). At 2 weeks, significantly more osseous tissue was formed after the systemic administration of simvastatin that was dissolved in water when this was compared with the results of using diclofenac (STS1 vs. DFS1, *p* < 0.001). On the contrary, after the histological evaluation of the 4th week’s samples, a reduced healing progress was observed for the groups with the systemic administration of simvastatin when it was compared to that of the natural bone healing group (STS2 vs. A2, *p* = 0.025). Finally, the systemic administration of diclofenac caused a statistically significant inferior bone formation when it was compared to that which was obtained by the systemic administration of simvastatin and in the natural bone healing group (DFS2 vs. STDS2, *p* = 0.04 and DFS2 vs. A2, *p* = 0.016).

Finally, the evaluation among the groups, where a collagen membrane with simvastatin dissolved in water was applied (STL), revealed that there was a statistically significant negative effect on bone formation when it was compared to the results of the natural bone healing group (STL1 vs. B1, *p* = 0.026, STL2 vs. B2, *p* = 0.009, STL3 vs. B3, *p* = 0.001) at all time spans and when it was compared to that which was obtained through administering simvastatin dissolved in DMSO at 4 weeks (STL2 vs. STDL2, *p* = 0.014). The percentage of bone formation where the membrane was imbued with simvastatin which was dissolved in DMSO (STDL) after 2 and 4 weeks was not significantly different when it was compared to that of the natural bone healing group (STDL1 vs. B1, *p* = 0.068, STDL2 vs. B2, *p* = 0.25), which showed quite a positive effect for the solvent DMSO, which reduced the release rate of simvastatin, and thus, limited the negative effect of the administered drug. However, this result was diminished after 8 weeks, where statistically higher bone formation was observed in empty cavities when this was compared with the defects that were covered by the DMSO-dissolved simvastatin-loaded collagen membrane (STDL3 vs. B3, *p* = 0.019).

## 4. Discussion

The need for increased bone formation after a tooth extraction or the application of regenerative techniques, as well as the pursuit of an increased rate of ossification around dental implants during osseointegration are some of the challenges that dentists are called upon to face in everyday clinical practice. NSAIDs and statins are two groups of drugs that affect bone healing and bone resorption, and these are being widely prescribed after dental interventions or are being taken on a regular basis by a large percentage of patients for other medical conditions. Scientific data on the effect of such medication on bone-healing mechanisms remain inconclusive, hence, the present study attempted to elucidate this effect in vivo. The safety and efficacy of drugs can be well tested on laboratory animals in order to detect their adverse effects as well as the appropriate dose before their administration on humans. The in vivo study protocol that was performed, which included a quite complicated interventional procedure, is recognized as the standard when studying materials with osteo-inductive properties and it represents a reliable and economical way of estimating new bone formation percentages [22].

The present research demonstrated a rather negative effect of the systemic administration of diclofenac. The laboratory rats of this group did not survive the period of 8 weeks of healing and thus, only values after the 2nd and 4th experimental week could be evaluated. The exhaustion and loss of weight of the laboratory animals were the primary reasons that led the researchers to evaluate the samples from the brain, liver, kidney, and muscles of the rats. A severe myopathy was observed in the histological samples that were taken from the quadriceps muscle, as well as inflammation which was recorded in the area of the drug injection. Diclofenac has been reported to have a direct toxic effect on the vascular endothelial cells in orthopedic research [23]. Inflammation was also observed in the present study during the histological evaluation of the samples that were taken from the deliberately induced bone defects. Those findings proved the toxic side effects of diclofenac and demonstrated its inhibiting role in osteogenetic activity. The latter is in accordance with data that are reported in the literature, where the long-term administration of diclofenac delayed the bone formation around the defects and resulted in an inflammatory reaction in the tissues [24,25]. The local inflammation that was observed in the initial phase of the bone healing may be attributed to the inhibiting role of diclofenac against COX-1 and COX-2, while the production of prostaglandins and the osteoblastic proliferation are also being inhibited by the administered medication, resulting, thus, in the discoordination of healing mechanisms. It has also been reported that diclofenac inhibits BMP-7 which induces the differentiation, maturation and proliferation of osteoblasts from mesenchymal cells [26]. Recently reported data state that NSAIDs could demonstrate a negative effect on the bone healing process by down-regulating chondrogenesis and endochondral ossification [27], and that diclofenac has a negative impact on pre-osteoblast cell growth [17]. Bissinger et al. [28] investigated the effect of diclofenac on fractures through a biomechanically, morphologically and by 3-dimensional microstructural analysis and reported an inhibiting effect on bone healing, but also additional changes which occurred in the microstructural bony network. The cytotoxic effect of diclofenac depends on the exposure to, time with, and concentration of the drug [29]. The exact mechanism though through which bone formation is being inhibited by diclofenac should be further investigated in histological studies on a cellular level.

As far as simvastatin is concerned, the experimental animals in the groups STS and STDS, where the drug was systemically administered, did not survive after the 4th week of the healing period. The animals presented a severe myopathy and inflammation in the injection area. The medication was injected intraperitoneally in order to achieve the better distribution of the substance in the bones [30]. The exhaustion that was observed in those animals led the researchers to study the local administration of the HMG-CoA reductase inhibitor, as well as a combination of it with the DMSO solvent in order to ensure the better dissolution, integration with and gradual absorption of simvastatin, and consequently, a possible reduction of systemic toxicity. However, the local administration of simvastatin (STL groups) did not enhance the bone formation in our investigation. It has been reported that statins do not promote the early osteoblastic differentiation of mesenchymal cells, and therefore, they do not appear to promote fracture healing, even if they are directly targeting the bone marrow cells [31]. In contrast to the latter, a recent meta-analysis proved that the local application of the drug improved the percentage of new bone formation [32], while its local administration has also been reported to stimulate and hasten osseous regeneration in RCTs [33]. In our study, a promotion of osteogenetic activity was recorded between the 2nd and 4th week of the healing period, only when the DMSO solvent was used in combination with the fleece membrane. Different carriers have been used to transfer statins to the healing area, such as calcium sulfate [34,35], methylcellulose gel [36,37], or gelatin sponge [38,39]. Locally applied simvastatin which was carried by calcium phosphate has been reported to be biocompatible, to enhance bone repair and to induce statistically greater bone formation than cloth or calcium phosphate can, alone [40]. In the present study, the fleece collagen membrane was applied, which has been successfully used to transfer NSAIDs to the bone healing area [26]. The osteogenetic effect of simvastatin has been reported to be greater when it is acting synergistically with other medication such as ezetimibe [41], hormones [42] or lasers [43], while nanofibers have also been lately used to transfer the drug to the affected area [41]. It appears that its local administration may have a significant impact on bone healing and should be further investigated with the type of carrier that plays a significant role in bone repair induction. Overall, the systemic administration of simvastatin was accompanied by severe systemic adverse effects on the laboratory animals. The effective dosage in rats to achieve bone formation has been reported to be high, while increasing the dose may be accompanied by adverse effects, such as muscle, liver, and renal damage [44]. Furthermore, statin associated myotoxicity has been recently proved with muscle adverse effects ranging from myalgia to potentially fatal rhabdomyolysis [45,46]. In groups where simvastatin was locally administered, a promotion in the osteogenetic activity was recorded only after combination with the DMSO solvent, which reduces the toxicity of the HMG-CoA reductase inhibitor. Clinicians should be aware of the toxicity of statins when they are prescribing such medication or when they are performing surgical interventions on patients that systemically take statins for other medical conditions to enable informed treatment decisions or treatment modifications, if appropriate. An interesting finding of the present study was the superior bone healing that was observed for group A where no intervention was performed, compared to group B where the collagen membrane was applied. Natural bone healing proved to be a more successful route, with a steady increase in bone density in all of the control groups. The bone healing pace evolved without any significant differences after 4 and 8 weeks of healing for group B, i.e., after the beginning of the absorption of the collagen membrane. It appears that the natural healing of bone defects without interventions (group A) is favorable to bone formation.

The present research was performed on animals and its results cannot be fully extrapolated to humans, which is a limitation. Since there were several experimental groups, the investigators chose not to create further subgroups with different dosage of the administered medication. The effect of the dosage of the medication could also affect bone formation, and this should be further investigated. Micro computed tomography could also be applied to give more reliable 3-D quantitative data of the bone regeneration within the created bone defects. Furthermore, different routes of drug administration were tested and the result in bone formation was statistically analyzed, but there was no further evaluation of the outcomes on a cellular level. The exact mechanisms through which statins and NSAIDs affect the healing process should be further evaluated in histomorphometric studies.

## 5. Conclusions

The systemic administration of simvastatin results in bone loss and severe systemic, adverse effects on animals. The local administration of simvastatin in combination with the DMSO solvent on deliberately induced bone defects enhanced bone formation between the 2nd and 4th week of the healing period. The use of a DMSO solvent appears favorable since the solvent allows for the better dissolution and gradual absorption of the drug and reduces the level of systemic toxicity. The systemic administration of diclofenac has a negative effect on bone healing. Clinicians should take these findings into consideration when they are prescribing drugs after surgical interventions and choose among medications that do not prohibit bone healing.

## Figures and Tables

**Table 1:** Groups’ classification. Seven groups were created according to route of drug administration and type of drug. Eighteen subgroups created according to experiment time span. Sample size (n) is inhomogeneous among groups where animals died due to toxicity of the administered medication or due to the destruction of the samples during preparation for histological analysis.

Test Groups	Experiment Time Span: 2 Weeks	Experiment Time Span: 4 Weeks	Experiment Time Span: 8 Weeks
A (n = 33)	A1 (n = 16)	A2 (n = 9)	A3 (n = 8)
B (n = 35)	B1 (n = 14)	B2 (n = 12)	Β3 (n = 9)
STS (n = 27)	STS1 (n = 14)	STS2 (n = 13	-
STL (n = 29)	STL1 (n = 13)	STL2 (n = 8)	STL3 (n = 8)
STDS (n = 20)	STDS1 (n = 10)	STDS2 (n = 10)	-
STDL (n = 31)	STDL1 (n = 9)	STDL2 (n = 13)	STDL3 (n = 9)
DFS (n = 20)	DFS1 (n = 12)	DFS2 (n = 8)	-

## Data Availability

Not applicable.
